# Ki-67 expression in anal intraepithelial neoplasia in AIDS

**DOI:** 10.1590/S1516-31802001000300007

**Published:** 2001-05-02

**Authors:** Edenilson Eduardo Calore, Carmen Ruth Manzione, Sidney Roberto Nadal, Maria José Cavalieri, Nilda Maria Perez Calore, Regina Paes dos Santos

**Keywords:** Acquired Immunodeficiency Syndrome, Condylomata accuminata, Ki-67, Anal intraepithelial neoplasia (AIN), HPV, Síndrome da Imunodeficiência Adquirida, Condilomas acuminados Ki-67, Neoplasia intraepitelial anal (NIA), HPV

## Abstract

**CONTEXT:**

AIDS is one of the most important risk factors for progression and recurrence of anogenital condyloma. In a previous work, we observed that patients with warts and high-grade AIN (HAIN) had recurrences more frequently than did patients with warts without AIN. The mechanisms of this increased incidence of high-grade lesions in AIDS are not known.

**OBJECTIVE:**

We studied the expression of the proliferative marker Ki-67 by immunohistochemical methods, in specimens of anal condyloma from HIV+ patients to clarify whether its expression can be associated to the grade of AIN.

**DESIGN:**

A retrospective study of hiltological specimens.

**SETTING:**

University referral unit.

**SAMPLE:**

34 patients were divided into two groups: (1) condylomas with low grade AIN (LAIN), with 25 patients; and (2) condylomas with HAIN, with 9 patients. In this latter group we examined two areas: 2A (HAIN area) and 2B (LAIN area).

**MAIN MEASUREMENTS:**

The immunohistochemical reaction for Ki-67 was done on histological sections. Slices were lightly stained with hematoxylin, to help us in Ki-67 positive cell counting. The percentage of Ki-67 marked nuclei was calculated. We applied one-way variance analysis for statistics.

**RESULTS:**

The mean number of Ki-67 positive cells in group 1 was 19.68 ± 10.99; in group 2 (area A) it was 46.73 ± 10.409; and in area B it was 36.43 ± 14.731. There were statistical differences between groups 1 and 2A and between groups 1 and 2B. Ki-67 positive cells predominated in the lower layer in LAIN. Positive Ki-67 cells were found in all layers in group 2A, and in group 2B they predominated in the two lower or in all layers of the epithelium.

**CONCLUSIONS:**

Our results suggest that LAIN areas (using routine staining techniques) in HAIN can have a biological behavior more similar to HAIN.

## RESUMO

**CONTEXTO:**

A síndrome da Imunodeficiência Adquirida (SIDA/AIDS) é importante fator de risco para a progressão e recidiva dos condilomas anais. Em estudo anterior, nós observamos que os doentes com condilomas anais associados AIN de alto grau (HAIN) recidivaram mais que aqueles sem essa lesão, embora todos tivessem sido tratados de maneira similar.

**OBJETIVO:**

No presente trabalho, estudamos a expressão do Ki-67 em condilomas acuminados e lesões pré-neoplásicas da região perianal de doentes HIV+, visando saber se essa expressão pode se correlacionar com o grau de AIN (HAIN ou LAIN).

**LOCAL:**

Centro de Referência Universitário.

**AMOSTRA:**

34 pacientes divididos em dois grupos: (1) condilomas com AIN de baixo grau (LAIN)-25 doentes; e (2) condilomas com HAIN- 9 casos. Nesse grupo, examinamos duas áreas distintas em cada corte: 2A (área com HAIN) e 2B (área com LAIN).

**TESTE DIAGNÓSTICO:**

Realizamos reação imunohistoquímica para Ki-67 nos cortes histológicos. As lâminas foram coradas fracamente com hematoxilina para auxiliar na contagem dos núcleos. Calculamos a porcentagem de núcleos Ki-67 positivos. Aplicamos a análise da variância na avaliação estatística.

**RESULTADOS:**

A porcentagem média de células Ki-67 positivo no grupo 1 foi 19, 68 ± 10, 99; no grupo 2A foi 46, 73 ± 10, 409 e no grupo 2B foi de 36, 43 ± 14, 731. Observamos diferença estatística entre os grupos 1 e 2A e os grupos 1 e 2B. As células Ki-67 positivo predominaram no terço inferior do epitélio nas LAINs, e em todas as camadas nos casos de HAIN. Já, no grupo 2B ocorreram nos dois terços inferiores ou em todo o epitélio.

**CONCLUSÕES:**

Nossos resultados sugerem que as regiões com aspecto histológico de LAIN (pelas técnicas rotineiras de coloração), em tecidos com HAIN, podem apresentar comportamento biológico semelhante às áreas com HAIN.

## INTRODUCTION

Anal condylomata accuminata incidence has been increasing since the AIDS epidemic began,^1^ suggesting that immunodepression is an important factor in this appearance and recurrence.^2, 3^ Moreover, this group of patients tends to develop pre-malignant anal lesions, known as anal intraepithelial neoplasia (AIN), mainly among male homosexuals.^4^ In addition to this, we noticed in another study that patients with high-grade AIN (HAIN) had more wart recurrence than patients with condylomas without HAIN.^5^

Factors involved in AIN development and the high incidence of condyloma recurrence in these patients are still not completely clarified. The high incidence of human papillomavirus (HPV) in anogenital lesions of HIV+ individuals is not enough to explain the frequent appearance of AIN in this group of patients.^6^ Some authors believe that other infectious agents could provoke AIN and anal carcinoma. Palefsky et al.^7^ observed anal carcinoma associated with Herpes virus simplex (HVS), but no etiological relationship could be proven.

Cell proliferation can be followed up by the expression of some cell proteins. Thus, the expression of Ki-67, a cell proliferation marker, is one of the most feasible methods for establishing the cell proliferative potential in neoplastic lesions of some organs, including pre-neoplastic lesions in the uterine cervix. Ki-67 expression appears at the beginning of the S phase, attains its highest level during mitosis and decreases in the G1 phase. When compared to other cell markers, Ki-67 is more efficient than c-myc protein, which presents positivity only for invasive carcinomas and HAIN from cervical biopsies, whereas Ki-67 expression has been noticed in pre-neoplastic lesions, distinguishing HAIN and LAIN (low-grade).^8^ It has also been observed that Ki-67 expression increases in AIN, from routinely processed cervical biopsies, when compared to squamous metaplasia and normal cells.^9^ In a comparative study, Ki-67 expression was better than p53 protein expression, which could be rarely detected in epithelial cell proliferation evaluation in uterine cervical cancer^10^ or in male genital warts.^11^

In this article, we have studied Ki-67 expression in anal condylomata accuminata and anal pre-neoplastic lesions from HIV+ patients to clarify whether its expression can be correlated with the grade of AIN (HAIN or LAIN).

## METHODS

This was a retrospective study, in which we utilized paraffin blocks of anal condylomas removed from HIV+ patients. We selected 36 specimens sent for histological examination from 1995 to 1998. The inclusion criteria were AIDS patients submitted to surgical treatment for anal condylomata accuminata, no use of antiretroviral agents and no associated anal disease. We made slides on which transversal sections including the basal membrane and epithelium were observed. Two patients were excluded because their paraffin blocks did not permit an examination of all the layers of the epithelium.

Hematoxylin-eosin (HE) staining, made at the outset, revealed 25 cases of condylomas with LAIN, which were named Group 1, and nine cases of condylomas with HAIN, named Group 2. In Group 2 we counted Ki-67 positive cells in areas with HAIN (group 2A) and in regions without HAIN of the same condylomas (group 2B).

The ages in Group 1 varied from 21 to 50 (mean 29.8 years), and in Group 2 the mean age was 34.8, ranging from 22 to 45 years. There was only one woman in each group.

The immunohistochemical reaction for Ki-67 was done on histological sections according to Hsu et al.^12^ The antibody used was anti-Ki-67 anti-human from rabbits (DAKO) at 1:300 dilution. Tissue slides were lightly stained with hematoxylin, coloring nuclei in blue, helping us to differentiate Ki-67 positive cells, which were brown.

We counted positive and negative nuclei from selected areas with the help of an Image Analysis System. Areas with the most positivity for Ki-67 were selected for counting (500 cells for each sample). The percentage of Ki-67 marked nuclei was calculated. We applied the one-way variance analysis for statistics.

## RESULTS

### Ki-67 stain pattern

Diffuse nuclear-stain or dot-stain of variable intensity was observed in both groups. Each positive nucleus was counted, regardless of the staining pattern.

### Distribution of Ki-67 positive cells in layers of the epithelium

Ki-67 positive cells predominated in the lower layer in group 1, and in all layers or in the two lower layers in group 2. In areas with LAIN of condylomas with HAIN (Figure 2B), Ki-67 positive cells appeared in the two lower layers of the epithelium in five cases, and they were distributed throughout all layers in four cases.

### Percentage of Ki-67 positive cells

**Figure 1:**
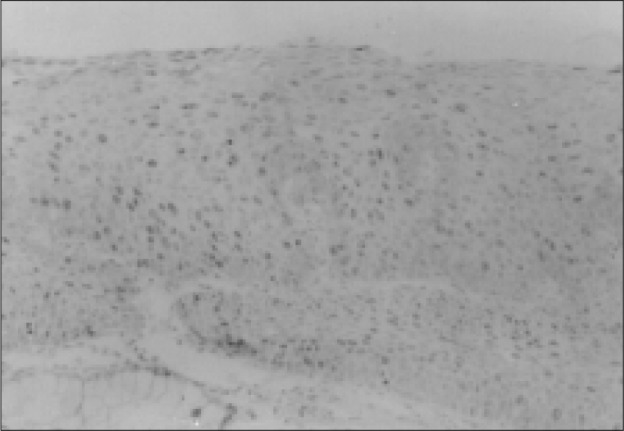
In this figure many Ki-67 positive cells can be seen in all layers of the epithelium in a case of HAIN from an anal verge biopsy. (X225).

**Figures 2A and 2B:**
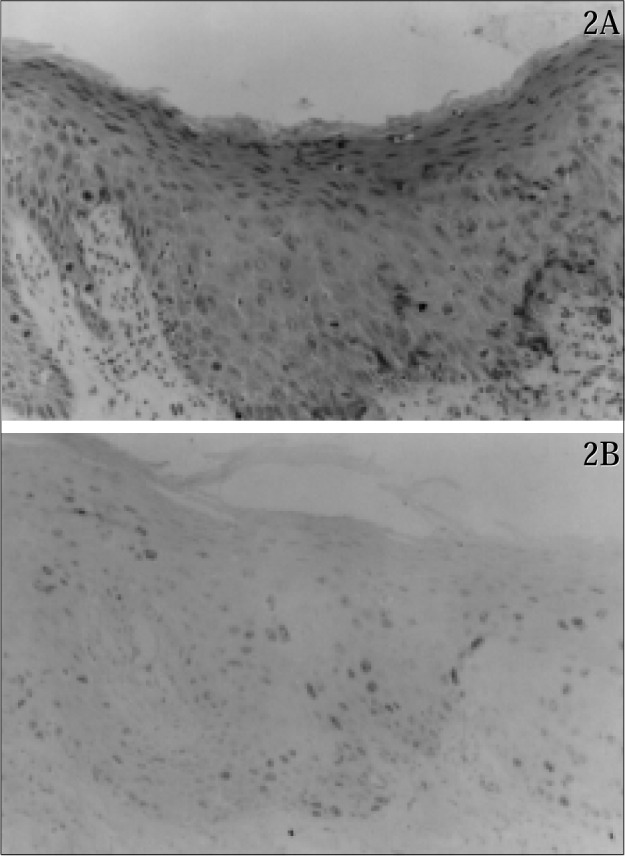
Serial sections stained respectively with HE, and immunostained with Ki-67, in an area of LAIN, in a case of HAIN. 2A- Area of the epithelium with LAIN appearance upon HE staining. 2B- In a serial section, there are a great number of Ki-67 positive cells in all layers of the epithelium. (X225).

The average number of Ki-67 positive cells in group 1 was 19.68 (EP 10.99), in group 2A it was 46.73 (EP 10.409), and in 2B it was 36.43 (EP 14.731) (Figures 1, 2A and 2B). Statistical differences between groups 1 and 2A and groups 1 and 2B were significant (P = 0.00), and there was no significance between groups 2A and 2B. (P = 0.576)

## DISCUSSION

We included only patients that did not received antiretroviral drugs, because the several therapeutic schemes and protease inhibitors introduced after 1996, changing systemic immunity, could have modified our results. We excluded two patients whose slides did not permit the identification of all layers of the epithelium.

Some authors have suggested an alarming incidence of dysplasia in condylomas from male homosexuals, especially when they are HIV positive.^4,5^ Some others have shown a high frequency of HPV infection, as with pre-neoplastic lesions in cervical epithelium in HIV positive women.^13–17^ Moreover, HIV+ women have more risks for cervical cancer than HIV negative women.^18,19^

Mechanisms related to the development of AIN are still not well understood. High-grade cervical intraepithelial neoplasia is strongly associated with HPV types 16 and 18 and less frequently with HPV types 31, 33, 35 and 51. These types of HPV have been detected in 50 to 90% of genital neoplasia.^20,21^ In low grade cervical intraepithelial neoplasia, the most frequent types are 6 and 11, considered to present low risk of malignancy.^7,21^ Nevertheless, the reasons for the high incidence of AIN and for the elevated frequency of recurrence in anal condylomas from HIV + patients are unknown.

We confirmed in our study that there were more Ki-67 positive cells in condylomas with HAIN than in warts with LAIN. But the most important result of this work was the increased number of Ki-67 positive cells in the non-HAIN areas of condylomas with HAIN. This finding suggests that these regions could have the same biological behavior as HAIN, despite their benign aspect in routine HE staining. The same was true for Ki-67 positive cell distribution in the different layers of the epithelium. We noticed these cells in all layers in HAIN (2A) and in the lower two-thirds of the epithelium in nonHAIN areas of the same section. This fact reinforces the hypothesis and helps us to explain the recurrence tendency in condylomas with HAIN. Nevertheless, factors like promiscuity and a new HPV infection cannot be excluded.

Ki-67 is one of the most sensitive proliferative cell markers for genital epithelium.^9^ The increased index of cell proliferation observed in our cases of AIN may reflect the ease of incorporation of the HPV DNA to the epithelial cell genome.

These results surprised us because they occurred in spite of determining more Ki-67 expression in condylomas with HAIN than in those with LAIN. This method allowed us to show that areas of condylomas with HAIN with a benign aspect, in routine stains, had more Ki-67 positive cells than non-HAIN condylomas.

## References

[1] Croxson T, Chabon B, Rorat E, Barash IM (1984). Intraepithelial carcinoma of the anus in homosexual men. Dis Colon Rectum.

[2] Laurent R (1996). Genital papillomavirus infections. Rev Prat.

[3] Penn I (1986). Cancers of the anogenital region in renal transplant recipients. Analysis of 65 cases. Cancer.

[4] Metcalf AM, Dean T (1995). Risk of dysplasia in anal condyloma. Surgery.

[5] Nadal SR, Calore EE, Manzione CR, Galvão VM (1996). Seguimento pós-operatório de condilomas acuminados perianais em doentes HIV+. Rev Bras Coloproct.

[6] Van Landuyt H, Mougin C, Drobacheff C (1993). Anogenital papillomavirus lesions in humans with or without HIV infection. Comparison of colposcopic, histopathological and virological results. Ann Dermatol Venereol.

[7] Palefsky JM, Holly EA, Gonzales J (1991). Detection of human papillomavirus DNA in anal intraepithelial neoplasia and anal cancer. Cancer Res.

[8] Devistor B, Bonnier P, Piana L (1993). C-myc protein and Ki-67 antigen immunodetection in patients with uterine cervix neoplasia: correlation of microcytophotometric analysis and histo-logical data. Gynecol Oncol.

[9] Al Saleh W, Delvenne P, Greimers R (1995). Assessment of Ki-67 antigen immunostaining in squamous intraepithelial lesions of the uterine cervix. Correlation with the histologic grade and human papillomavirus type. Am J Clin Pathol.

[10] Bar JK, Harlozinska A, Markowska J, Nowak M (1996). Studies on tumor proliferation using monoclonal antibody, Ki-67 and expression of p53 in cancer of the uterine cervix. Eur J Gynaecol Oncol.

[11] Ranki A, Lassus J, Niemi KM (1995). Relation of p53 tumor suppressor protein expression to human papillomavirus (HPV) DNA and to cellular atypia in male genital warts and in premalignant lesions. Acta Derm Venereol.

[12] Hsu SM, Raine L, Fanger H (1981). The use of antiavidin antibody and avidin-biotin peroxidase complex in immunoperoxidase techniques. Am J Clin Pathol.

[13] Calore EE, Cavalieri MJ, Shirata NK, Araújo MF (1995). Papillomavirus in cervicovaginal smears of women infected with Human Immunodeficiency Virus. S Paulo Med J.

[14] Feinglod AR, Vermund SH, Burk RD (1990). Cervical cytological abnormalities and Papillomavirus in women infected with human immunodeficiency virus. J Acq Immun Def Synd.

[15] Johnson JC, Burnet AF, Willet GD, Young MA, Doniger J (1992). High frequency of latent and clinical human Papillomavirus cervical infections in immunodeficiency virus-infected women. Obstet Gynecol.

[16] Maiman M, Fructher R, Cerur E (1993). Recurrent cervical intraepithelial neoplasia in HIV seropositive women. Obstet Gynecol.

[17] Scheafer A, Friedman W, Mielke M, Schwartlander B, Koch MA (1991). The increased frequency of cervical dysplasia-neoplasia in women infected with the HIV virus is related to the degree of immunosuppression. Am J Obstet Gynecol.

[18] Calore EE, Cavalieri MJ, Calore NMP (1998). Squamous intraepithelial lesions in cervical smears of human immunodeficiency virus-positive adolescents. Diag Cytopathol.

[19] Schrager LK, Friedland GH, Maude D (1989). Cervical and vaginal squamous cell abnormalities on women infected with human immunodeficiency virus. J Acq Immun Def Synd.

[20] Syrjanen SM, von Krogh G, Syrjanen KJ (1987). Detection of human papillomavirus DNA in anogenital condylomata in men using in situ DNA hybridization applied to paraffin sections. Genitourin Med.

[21] Wells M, Griffiths S, Lewis F, Bird CC (1987). Demonstration of human papillomavirus types in paraffin processed tissue from human anogenital lesions by in-situ DNA hybridization. J Pathol.

